# Hematological and biochemical status of Beta-thalassemia major patients in Bangladesh: A comparative analysis

**Published:** 2016-01-01

**Authors:** Md. Fazlul Karim, Md. Ismail, AKM Mahbub Hasan, Hossain Uddin Shekhar

**Affiliations:** Department of Biochemistry and Molecular Biology, University of Dhaka, Dhaka-1000, Bangladesh

**Keywords:** Beta-thalassemia major, Liver function, Thyroid function, Hematological characteristics

## Abstract

**Background:** Thalassemia is one of the most common hereditary disorders and Beta-thalassemia major is its severe form. The present study is concerned with the analysis of liver function, thyroid function and estimation of critical serum ions as well as hematological characteristics in beta-thalassemia patients and controls.

**Subjects and Methods:** The study included 54 patients with beta-thalassemia major and 54 healthy individuals matched by sex and age. The activity of Alanine transaminase (ALT), Alkaline phosphatase (ALP) and Aspartate transaminase (AST) were assessed in order to evaluate the liver function. Serum content of iron (Fe), calcium (Ca), magnesium (Mg), sodium (Na) and potassium (K) were also estimated. Tri iodothyronine (T3), Thyroxin (T4) and Thyroid-stimulating hormone (TSH) levels were assessed in order to evaluate the thyroid function. Hemoglobin (Hb), ferritin, hematocrit (HCT), mean corpuscular volume (MCV), mean corpuscular hemoglobin (MCH), mean corpuscular hemoglobin concentration(MCHC), total iron binding capacity (TIBC) and creatinine level were also measured.

**Results:** Significantly, higher ALT (P< 0.001), AST (P< 0.05), ALP (P< 0.001) activities and lower creatinine (P< 0.001) level in beta-thalassemia patients were found in comparison to healthy individuals. Lower serum level of calcium (P< 0.05), magnesium (P< 0.05) and higher level of iron (P> 0.05), sodium (P> 0.05) and potassium (P > 0.05) have been found in patients in comparison to healthy individuals. Hematological parameters like Hb (P< 0.001), ferritin (P< 0.05), HCT (P< 0.001), MCV (P< 0.05) and MCH (P< 0.05) have been significantly reduced in patients except MCHC (P> 0.05). No significant difference was observed in thyroid function between patients and control group.

**Conclusions:** Our study demonstrates that beta-thalassemia patients and controls have difference in liver function, thyroid function, serum contents of ions and hematological characteristics.

## Introduction

 Thalassemia is a group of inherited hemoglobin disorders characterized by reduced synthesis of one or more of the globin chains leading to imbalanced globin synthesis which is the major factor in determining the severity of the disease in the thalassemia syndromes. Beta-thalassemia results from a defect in beta globulin chain production and ranges from clinically silent heterogeneous thalassemia minor to severe transfusion-dependent thalassemia major.^1^^,^^2^ Beta-thalassemia major is a very serious blood disorder since affected individuals are unable to make enough healthy red blood cells and that is why they are totally dependent on blood transfusion throughout their life.^3^ Various complications caused by this disease including growth retardation,^4^^-^^6^ endocrine dysfunction,^7^^,^^8^ hypothyroidism,^9^^,^^10^ progressive liver failure^11^ and abnormal kidney function. Trace metals, especially iron, are implicated as causative agents in excessive generation of free radicals which are capable of causing oxidative damage to erythrocytes.^12^ Iron metabolism in human is unidirectional because of being unable to be eliminated by the excretory route. Therefore, excess of iron is deposited in vital organs such as heart, liver, spleen and endocrine organs.^13^^-^^15^ Estimation of calcium, sodium, potassium and magnesium is also valuable. Several authors have reported a high incidence of endocrine abnormalities in children, adolescents and young adults suffering from thalassemia. However, the incidence of endocrinopathies varies among different series of the patients. Trace minerals have been shown to have influence on growth and hormones e.g. zinc deficiency is considered a causative factor in osteoporosis and endocrinopathies.^16^^,^^17^ The aim of this study is to evaluate the liver function parameters, thyroid function parameters and hematological features in beta-thalassemia patients and compare them with the control group consisted of Bangladeshi children.

## SUBJECTS AND METHODS

**Subjects**

 A total of 54 beta-thalassemia patients (27 male and 27 female) and 54 controls (27 male and 27 female) were included in this study. Beta-thalassemia patients were aged between 3 and 12 years old. The control group consisted of healthy children with ages ranging from 5 to 12 years. Patients were interviewed by trained personnel (interviews were conducted with parents of infants but the others were interviewed directly) using a standardized questionnaire. Participants had haematological evidence of beta–thalassemia such as profound hypochromic anaemia, mean erythrocyte volume less than 75 fl, electrophoretic demonstration of haemoglobin A2 ( higher than 3.5% of total haemoglobin) Meanwhile, both parents had beta-thalassemia. The presence of the disease was also evaluated by genetic analysis which confirmed the absence or the reduced levels of alpha- or beta-chain synthesis in haemoglobin. Patients were recruited from Bangladesh Thalassemia Hospital, Green road, Panthapath, Dhaka, Bangladesh. Patients with thalassemia intermedia and minor were excluded from the study. All patients were maintained on a regular transfusion program and received deferoxamine (DFO) as a chelating agent. Informed consent was obtained from the patients and they were given the right to withdraw from the study at any time. The study protocol was approved by the Human Ethics Committee of Dhaka University.

**Sampling and data collection**

As per standard procedures, anthropometric measurement such as height and weight were collected. An integrated questionnaire was employed in order to obtain information on age and education. The data and blood samples were collected over a period of 10 months (from September 2012 to June 2013). Under sterile conditions, about 5 mL of blood was collected into EDTA vacutainers through venipuncture. An aliquot of blood (2.0 mL) was taken, transferred to another tube, mixed thoroughly and stored at -20^o^C until completion of blood count. The tube containing the remaining 3 mL blood sample was then centrifuged for 15 min at 3,000 rpm and plasma samples were collected in Eppendorf tubes using Pasteur pipette. For further use, the sample was stored at 20^o^C until further use.

**Biochemical assays**

AST, ALT and ALP activities were assessed using commercial kits according to the manufacturer’s instructions (Vitro Scient, Egypt). Creatinine level was measured by using commercial kit according to the manufacturer’s instructions (Vitro Scient, Egypt). Thyroid function was assessed by measuring serum level of T3, T4 and TSH using commercial quantitative ELISA kits and procedures of Sigma-Aldrich, Germany. Serum iron and calcium were assessed by using commercial kits according to the manufacturer’s instruction (Sigma-Aldrich and Biovision, respectively). Serum level of Mg was determined using the kits and procedures of Pro Dia International UAE. Finally, data were read using clinical chemistry analyzer (RA-50, Bayer Diagnostics and Ireland). Hemoglobin was colorimetrically determined applying the method of Van Kampen and Zijlstra.^18^ Hematological characteristics were assessed based on the laboratory protocol.^19^ Serum TIBC was measured according to the Ramsay’s Dipyridyl Method.^20^ Serum Na and K level were determined according to the RD. Mazzachi method.^21^ Serum Ferritin level was measured by the method of Jacobs et al.^22^

**Statistical analysis**

Statistical analysis was carried out using SPSS 16.0 and p-values were set at 0.05. Data are presented as Mean ± SD (standard deviation). The results were analyzed using analysis of variance (ANOVA) followed by the independent t-test. Correlations were calculated by the Pearson’s correlation coefficients.

## Results

 The mean age of beta-thalassemia major patients and controls is 7.8 and 8 years, respectively. Mean height in patients is 84.2 cm and in controls is 86.5 cm. Mean weight is 18.4 kg and 19 kg in patients and controls, respectively. The mean age at the start of transfusion was 4.3 years. Mean duration of blood transfusion is 7.1 years. Mean transfusion interval was 12 days. Pre-transfusion hemoglobin is 7.2 ± 1.5 (g/dL). There is statistically significant difference in Total Iron Binding Capacity (TIBC) between patients (163 ± 69.6 µg/dL) and controls (237.19 ± 56.28 µg/dL).

**Table 1:** Liver function test in Beta-thalassemia major patients (n=54) and controls (n=54)

**Parameters ** **(Unit)**	**Beta-thalassemia** **(n=54)** **Mean ± SD**	**Control** **(n=54)** **Mean ± SD**	**P-value**
**ALT (IU/L)**	81.5 ± 26.8	20 ± 5.7	P< 0.001
**ALP (IU/L)**	257.5 ± 51.1	136 ± 29.8	P< 0.001
**AST (IU/L)**	74.8 ± 21.7	16.3 ± 4.1	P< 0.05
**Creatinine ** **(µg/dL)**	0.4 ± 0.2	0.85 ± 0.26	P< 0.001

**ALT:** Alanine transaminase, **ALP:** Alkaline phosphatase, **AST:** Aspartate transaminase

The mean creatinine level (0.4 ± 0.2 µg/dL) was significantly lower (P< 0.001) than the controls (Table 1), indicating the renal dysfunction and requiring more advanced analysis. ALT, AST, ALP activities in beta-thalassemia patients are significantly different (P< 0.001, P< 0.05, P< 0.001, respectively) from controls. The evaluation of some essential trace element levels in beta-thalassemia major patients and controls are shown in Table 2.

As shown in Table 2, the serum iron value in patients was close to control values due to oral iron chelating drug .Moreover, no significant difference (P> 0.05) existed between the patients and controls, but the iron level was still found to be higher in patients compared to the controls. On the other hand, serum calcium and magnesium are significantly (P< 0.05) lower, while serum sodium and potassium are higher (non-significant, P> 0.05) in beta-thalassemia patients compared to controls. As shown in Table 3, there was a significant decrease in Hb (P< 0.001), HCT (P< 0.001), MCV (P< 0.05) and MCH (P< 0.05) values in beta-thalassemia patients compared to controls.

As shown in Table 4, all parameters of thyroid function (T3, T4 and TSH) were close to control values. The study included patients with ages ranging from 3 to 12 years. Hypothyroidism usually appears in the second decade of life and is thought to be associated with iron overload in patients with Beta-thalassemia major.^24^ All parameters of thyroid function in beta-thalassemia patients were not significantly different from those of controls.

## Discussion

 Beta-thalassemia, one of the most common genetic disorders in Asia,^33^^-^^36^ and most parts of the world, has already drawn the attention of scientific research. Thalassemia syndromes are a group of hereditary and severe disorders resulting from the homozygous state of one of the thalassemia or hemoglobin Lepore genes in infancy or childhood.^37^^,^^38^

It is accompanied with metabolic irregulation, iron overload; chronic hypoxia and cell damage.^38^ All physiological changes result in ineffective erythropoiesis, haemolysis and anaemia.^39^ Most patients are dependent on transfusion for their survival and bone marrow transplantation.

**Table 2:** Serum ions in Beta-thalassemia major patients (n=54) and controls (n=54)

**Parameters ** **(Unit)**	**Beta-thalassemia** **(n=54)** **Mean ± SD**	**Control** **(n=54)** **Mean ± SD**	**P-value**
**Fe (µg/dL)**	123 ± 40.5	110 ± 32	P> 0.05
**Ca (mg/dL)**	7.9 ± 0.6	8.5 ± 1.1	P< 0.05
**Mg (mg/dL)**	1.88 ± 0.2	2.2 ± 0.32	P< 0.05
**Na (mmol/L)**	138.8 ± 1.7	136 ± 2	P> 0.05
**K (mmol/L)**	5.2 ± 1.3	4.2 ± 0.9	P> 0.05

**Fe:** iron, **Ca:** calcium, **Mg:** magnesium, **Na:** sodium, **K:** potassium.

**Table 3:** Hematological characteristics in beta-thalassemia major patients (n=54) and controls (n=54)

**Parameters ** **(Unit)**	**Beta-thalassemia** **(n=54)** **Mean ± SD**	**Control** **(n=54)** **Mean ± SD**	**P-value**
**Hb (g/dL)**	7.2 ± 1.5	13 ± 1.4	P< 0.001
**Ferritin (μg/L)**	1249 ± 59 2	45 ± 17	P< 0.05
**HCT (%)**	21.5 ± 5.3	38 ± 6.2	P< 0.001
**MCV (μm3)**	70 ± 9.5	80 ± 11	P< 0.05
**MCH (pg)**	23.8 ± 3.8	28 ± 5	P< 0.05
**MCHC (g/dL)**	34.1 ± 2.8	36.7 ± 4.6	P> 0.05

**Hb:** hemoglobin, **HCT:** Hematocrit, **MCV:** mean corpuscular volume, **MCH:** mean corpuscular hemoglobin, **MCHC:** mean corpuscular hemoglobin concentration

**Table 4:** Thyroid function in beta-thalassemia major patients (n=54) and controls (n=54)

**Parameters ** **(Unit)**	**Beta-thalassemia** **(n=54)** **Mean ± SD**	**Control** **(n=54)** **Mean ± SD**	**P-value**
**T3 (ng/mL)**	1.3 ± 0.4	1.4 ± 0.3	P> 0.05
**T4 (µg/dL)**	8.8 ± 3.2	9.8 ± 4	P> 0.05
**TSH (µIu/mL)**	1.7 ± 0.9	2.5 ± 0.7	P> 0.05

**T3:** Triiodothyronine, **T4:** Thyroxine, **TSH:** Thyroid-stimulating hormone

Although an increasing number of patients are now treated with bone marrow transplantation, the majority of the patients still depend on regular transfusions. Regular transfusion and chelation therapy have improved the span and quality of their lives.^40^^,^^41^ In this study, beta-thalassemia patients have shown significantly low serum Ca levels (P< 0.05) in comparison with the controls. Similar results have been described in other studies^23^^,^^25^ which are in accordance with the findings of our study and these similarities have validated the outcomes of the present study. Low level of magnesium has been observed in this study and an explanation of hypomagnesemia is that it may occur due to lower thyroid hormones resulting from iron overload.^29^ Determination of sodium and potassium levels have revealed slightly higher sodium level and higher potassium level in beta-thalassemia patients in comparison to controls. These differences in serum content of sodium and potassium levels are not statistically significant (P> 0.05). These findings are similar with the outcomes of other scientific studies.^27^^,^^28^ An increased potassium level occurs in patients with red blood cell (RBC) hemolysis, which may occur in stored blood that is transfused to the patient since potassium tends to leak out of the RBC in stored blood.^27^ An increased sodium level in beta-thalassemia patients may be due to renal damage resulting from iron overload.^28^ Higher level of serum AST, ALT and ALP in beta-thalassemia patients indicate an abnormal muscle and liver function. These finding are in agreement with the finding of Maher Y. Abdalla et al.^24^ There is a positive correlation between serum ALT (r = 0.315) and AST (r = 0.291) concentrations and serum ferritin levels in beta-thalassemia patients compared to controls. These outcomes are similar to the finding of Maher Y. Abdalla et al.^24^ The high creatinine level indicates very low functioning capacity of the kidney though it needs further to substantiate the present study. All hematological parameters including Hb, HCT, MCV and MCH except MCHC were found to be significantly (P< 0.001, P< 0.001, P< 0.05, P< 0.05, respectively) lower than the controls, which are in accordance with the outcomes of the study conducted by Filiz Simsek et al.^31^.Total iron binding capacity (TIBC) was found to be significantly (P < 0.001) lower in beta-thalassemia patients than the controls. This result is similar with the result of Rahul A Ghone et al.^30^ Clinical data confirm that the decrease of the haemoglobin level is accompanied by a decrease in the number of erythrocytes and diminished values of their specific indexes (MCV, MCH, HCT, etc). An increase in serum iron and ferritin level in beta-thalassemia patients have been observed in this study, which is consistent with several other studies.^30^^-^^32^ In case of beta-thalassemia patients, absence of beta globin chains lead to accumulation of unpaired alpha globin chains. Excess presence of the alpha globin chains is a primary reason for the cellular oxidative damage and also iron overload.^30^ Higher ferritin content was directly linked to the accumulation of reactive iron in the tissues of these patients. Iron overload starts another pathological mechanism leading to oxidative damage of erythrocyte membranes, the so-called "second disease".^39^ In our study, there was a slight difference in thyroid function parameters and no significant difference was observed (P> 0.05).Our findings are in line with the outcome of Aamer Aleem et al.^23^ whose study has demonstrated that hypothyroidism is primarily a disease of the second decade of life. We are optimistic that present data will help developing a guideline regarding early diagnosis and mode of treatment in Bangladeshi population.

## CONCLUSION

 Presence of difference, in liver function, thyroid function, serum contents of ions and hematological characteristics, between beta-thalassemia patients and controls have been demonstrated in this study.
